# Therapeutic Approaches in COVID-19 Patients: The Role of the Renin-Angiotensin System

**DOI:** 10.1155/2022/8698825

**Published:** 2022-09-26

**Authors:** Farzaneh Ketabchi, Sina Jamzad

**Affiliations:** Department of Physiology, School of Medicine, Shiraz University of Medical Sciences, Shiraz, Iran

## Abstract

Two and a half years after COVID-19 was first reported in China, thousands of people are still dying from the disease every day around the world. The condition is forcing physicians to adopt new treatment strategies while emphasizing continuation of vaccination programs. The renin-angiotensin system plays an important role in the development and progression of COVID-19 patients. Nonetheless, administration of recombinant angiotensin-converting enzyme 2 has been proposed for the treatment of the disease. The catalytic activity of cellular ACE2 (cACE2) and soluble ACE2 (sACE2) prevents angiotensin II and Des-Arg-bradykinin from accumulating in the body. On the other hand, SARS-CoV-2 mainly enters cells via cACE2. Thus, inhibition of ACE2 can prevent viral entry and reduce viral replication in host cells. The benefits of bradykinin inhibitors (BKs) have been reported in some COVID-19 clinical trials. Furthermore, the effects of cyclooxygenase (COX) inhibitors on ACE2 cleavage and prevention of viral entry into host cells have been reported in COVID-19 patients. However, the administration of COX inhibitors can reduce innate immune responses and have the opposite effect. A few studies suggest benefits of low-dose radiation therapy (LDR) in treating acute respiratory distress syndrome in COVID-19 patients. Nonetheless, radiation therapy can stimulate inflammatory pathways, resulting in adverse effects on lung injury in these patients. Overall, progress is being made in treating COVID-19 patients, but questions remain about which drugs will work and when. This review summarizes studies on the effects of a recombinant ACE2, BK and COX inhibitor, and LDR in patients with COVID-19.

## 1. Background

The renin-angiotensin system (RAS) plays an important role in the regulation of the cardiovascular system. Angiotensin II (Ang II) is one of the main products of RAS produced from angiotensin I (Ang I) under the action of the angiotensin conversion enzyme (ACE). Ang II acts via the AT1 receptor, leading to vasoconstriction, direct and indirect reabsorption of sodium through the kidneys, releasing vasopressin and stimulating the hypothalamus's thirst center [1]. All these effects are essential in emergency conditions to maintain the blood flow of vital organs within normal limits. However, a high concentration of Ang II for a long period may cause cardiac hypertrophy and fibrosis, endothelial dysfunction, thrombosis, atherosclerosis, and arrhythmia [2]. Stimulating the AT2 receptor has the opposite effects of the AT1 receptor, like vasodilation and lowering blood pressure (BP) [3]. Ang II is converted into Ang 1–7 by ACE2, a transmembrane enzyme with carboxypeptidase terminal activity. Ang 1–7 acts through Mas receptors and has a counter-regulating action, leading to vasodilation and reduced parameters such as BP, cardiac hypertrophy, fibrosis, thrombosis, and arrhythmia [2–4].

**Figure 1:**
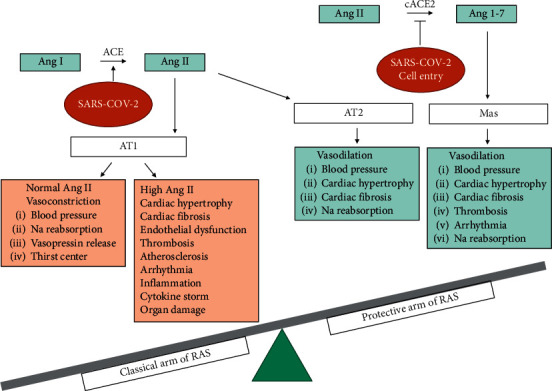
The imbalance in two arms of the renin-angiotensin system in COVID-19 infection: the classical arm vs. the protective arm.

**Figure 2:**
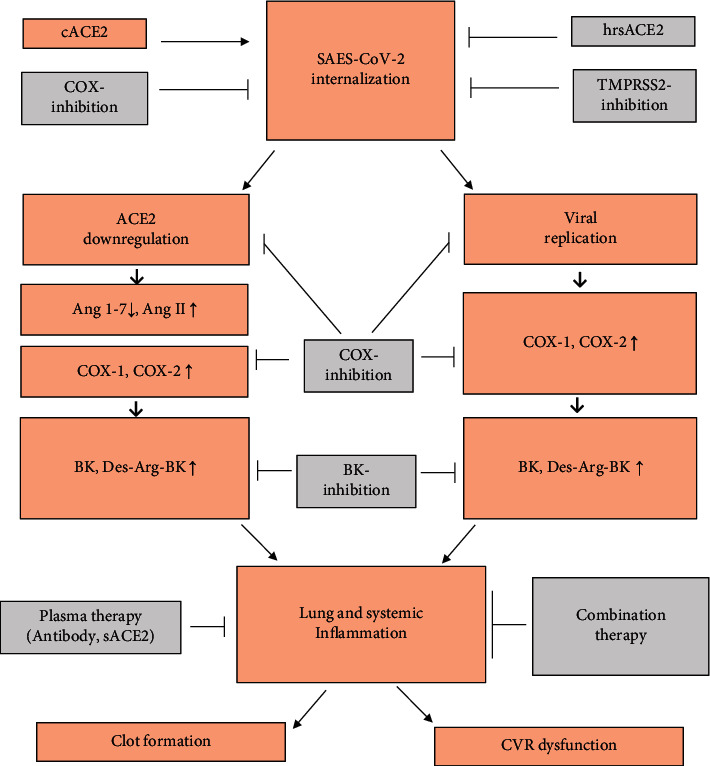
The relationship between sACE2, BK, and COX inhibitor, and plasma therapy in the patients with COVID-19. CVR: cardiovascular.

COVID-19 was first detected in China and quickly spread across the globe [5]. It is mainly characterized by cold symptoms that last for a few days. However, moderate to severe COVID-19 can be associated with acute pulmonary inflammation, cardiovascular failure, and coagulopathy. Despite large-scale vaccine programs and a variety of therapeutic approaches used in the treatment of COVID-19 patients, morbidity and mortality remain high. RAS plays an important role in inflammatory reactions, clot formation, and COVID-19-related virus infections [6–8]. The imbalance between the two arms of RAS (classical and protective arms) contributes to cytokine storm, hypercoagulability, and multiple organ damage in COVID-19 patients [8–11] (Figure 1). The pathogenesis of COVID-19 is related to a novel SARS coronavirus (SARS-CoV-2) that, like previous coronaviruses, enters host cells through ACE2 [12, 13]. Expressions of ACE2 have been described in many organs of the body, including the kidneys, fat tissue, the gastrointestinal tract, the heart, and airway epithelial cells [14–18]. ACE is a key enzyme for the inactivation of bradykinin (BK), while ACE2 breaks down the active metabolites of BK [19]. As a result, downregulation of ACE2 can accumulate active BK metabolites and worsen inflammatory reactions in patients with COVID-19. In addition, a relationship between cyclooxygenase (COX), a key enzyme of inflammation, and RAS has been suggested in COVID-19 patients [20]. A few studies also report the benefits of low-dose radiation (LDR) in the treatment of patients with COVID-19. However, radiation therapy can increase Ang II, the active metabolites of BK, and COX-2, which has an adverse effect on body tissues [21–24]. In this review, we discussed the studies related to the effects of recombinant ACE2, inhibitors of BK and cyclooxygenase (COX), and low-dose radiation (LDR), and their interactions with COVID-19 infection are summarized in Figure 2.

## 2. Interaction between ACE2 and SARS-CoV-2

Investigation of the interaction between coronaviruses and ACE2 in humans dates back to 2003 when it was reported that the SARS coronavirus (SARS-CoV) entered cells via ACE2 [25–27]. In 2004, the amino acid fragment of the virus's S protein that binds to ACE2 was identified [28]. Moreover, the outbreak of the disease in 2003–04 was shown to be lower than in 2002–03 due to the lower affinity of SARS-CoV protein to ACE2 protein [29]. In addition, it was demonstrated that SARS-CoV-2 can infect several cell types and immune cells, depending on the level of expression of ACE2 [30]. Several therapeutic approaches were recommended to reduce viral cell penetration or complications of the disease. Soluble recombinant human ACE2 (hrsACE2) was suggested to hide the SARS-CoV binding site, thereby preventing the virus from entering cells. [31]. In addition, a number of antibodies, peptides, and small compounds were introduced to slow viral replication, blocking the binding site of the S protein, or inducing conformation into the S protein [27, 32, 33]. However, a number of investigations were discontinued due to a decline in the incidence the disease in 2004.

## 3. Interaction between COVID-19 and ACE2

Two forms of ACE2 have been identified: cellular (cACE2) and soluble (sACE2). SARS-CoV-2 enters the cells through cACE2 and downregulates transmembrane protein [8]. Furthermore, the expression of ACE2 decreases in patients with COVID-19 [34]. There is a negative correlation between the ACE2 expression and the COVID-19 mortality rate [35]. The entrance of SARS-CoV-2 into host cells is blocked by serine protease TMPRSS2 inhibitors [36]. TMPRSS2 is shown to facilitate the entry of the virus by the S1 and S2 cleavages of SARS-CoV-2 [20, 37]. There are also other proteases that can play roles in SARS-CoV-2 internalization [37, 38]. Furthermore, the ADAM-17 protease releases ACE2 in a soluble form (sACE2) that circulates in the extracellular environment [39]. sACE2 has no membrane anchor used as a cell entry point for SARS-CoV-2 [8, 10]. Therefore, it is suggested to be a therapeutic target to prevent viral entrance in host cells. Studies on Vero cells and kidney organoids can confirm the role of hrsACE2 in preventing cell entry and replication of the virus [40, 41]. However, one study has raised the hypothesis that the effect of sACE2 on viral entry is dose-dependent: sACE2 with physiological concentration leads to viral entry through AT1 and vasopressin receptors, while, pharmacologic concentration may have an inhibitory effect [42]. Furthermore, the use of engineered extracellular vesicles (EVs) exposed to cACE2 and TMPRSS2 is demonstrated to be much more effective than the use of sACE2 for viral trapping and reduction of infection [43] (Table 1). It is important to mention that a high concentration of hrsACE2 is tolerated in ARDS patients without significant side effects [44]. As a result, a high concentration of hrsACE2 may influence COVID-19 patients with ARDS. In a case report study, an intravenous infusion of hrsACE2 twice daily for seven days was well tolerated in a 45-year-old woman with COVID-19. The patient survived until she was discharged on day 57 [45] (Table 2). There are also some review papers proposing the treatment of COVID-19 patients with hrsACE2 [9, 10, 46–48]. Plasma from patients who have recovered from COVID-19 may be an excellent source of neutralizing antibodies against the virus [49]. Soluble ACE2 has also been detected in plasma and may be of value in predicting COVID-19 outcomes [50]. Depending on the concentration of sACE2, sera from highly exposed uninfected subjects could more effectively neutralize SARS-CoV-2 infection in cellular assays, even in the absence of sufficient anti-CoV-2 IgG antibodies [51]. However, additional clinical studies are necessary to explore the effect of sACE2 as a promising therapeutic target on patients with COVID-19.

The downregulation of cACE2 increases the impact of ACE and Ang II in the body of COVID-19 patients. A cohort study showed that Ang II increases in the blood of patients suffering from COVID-19 [52]. It has been reported that hospitalized hypertensive COVID-19 patients who use ACE or AT1 antagonists had a lower risk of mortality than the others [53, 54]. Furthermore, COVID-19 patients with hypertension treated by Ang II receptor inhibitors are less likely to develop severe lung disease [55]. On the contrary, other studies did not show a difference between using ACE or AT1 inhibitors and COVID-19 severity markers [56–59]. In addition, a case-population study has indicated that the administration of RAS inhibitors does not increase the risk of COVID-19 for admission to the hospital and intensive care unit [60]. Moreover, there is no correlation between the administration of RAS inhibitors and the mortality rate in COVID-19 patients [61].

Other researchers also believe that treatment with RAS inhibitors should not stop in COVID-19 infections [62]. In general, the effects of RAS inhibitors on COVID-19 may be affected by the complexity of the pathophysiology of the disease. Furthermore, studies suggest that increased Ang II in a hypoxic environment may activate cancer pathways and tumorigenicity in body tissues, which should be considered in the future follow up of severe COVID-19 patients [63].

## 4. Interaction between COVID-19, BK, and ACE2

BK is an active polypeptide released by the kinin-kallikrein system (KKS) from damaged tissues. BK is produced from kininogens through kallikrein enzymes and converts to Des-Arg-BK (DABK) and other metabolites by kininase I and kininase II (ACE). DABK is one of the active metabolites of BK, which is hydrolyzed by ACE2 [19, 64–67]. BK and DABK may cause local vasodilation or vasoconstriction through B2 and B1 receptors, whereas both of them may decrease the mean systemic blood pressure. Mechanisms are related to species, vessels, and their downstream signaling pathways such as NO, cyclooxygenase (COX) products, and prostaglandins [68–70]. There are some interactions between RAS and KKS within the cardiovascular system. ACE inhibitors increase the blood levels of BK and Ang 1–7. BK may potentiate the effect of Ang 1–7 in the cardiovascular system and lead to vasodilatation and decreased BP [71]. The mechanism of this interaction can be related to the generation of NO [72]. In contrast, stimulation of B2 receptors potentiates the constrictive effect of the AT1 receptor on small mesenteric vessels in endotoxemia. This finding suggests the presence of AT1/B2 receptor heterodimers that lead to a strong contractile response to BK and Ang II [73].

KKS is a major component of inflammatory reactions and intrinsic coagulation pathways [74]. Inhibition of ACE2 during inhalation of endotoxin increases BK axis activity, neutrophil infiltration, and severe inflammation in the mouse lung [67]. BK is indicated to induce lung damage in ischemia-reperfusion and inflammation caused by parainfluenza-3 [75, 76]. A significant increase in BK and DABK increases vascular permeability, inflammatory reactions, and lung injury, leading to a serious illness called BK storm [77–79]. In addition, both Ang II and KKS stimulate plasminogen activator inhibitor-1 and clot formation, while Ang 1–7 has anti-inflammatory and antithrombotic effects [77, 80]. The relation between BK and COX activity has been reported in several experimental contexts. Inhibitions of the B2 and COX-2 receptors have an additive effect in reducing tissue damage [81, 82]. Therefore, these combination therapies can be useful for patients with COVID-19.

Alveolar epithelial cells express transcripts encoding proteins that play essential roles in the regulation of the KKS, RAS, and coagulation system [83]. In one case-control study, it was reported that the use of the icatibant B2 antagonist improved oxygenation in COVID-19 patients [84]. Furthermore, one randomized clinical trial reported that icatibant and Cle/kallikrein reduce the complications of COVID-19 and the duration of hospitalization (Table 2) [85]. Also, the administration of recombinant neprilysin, as an alternative ACE-2/Ang 1–7/Mas receptor axis, has a higher activity than ACE to BK degradation, and suggest for the treatment of COVID-19 patients [86]. Also, one of the side effects of ACE inhibitors, coughing, is associated with BK accumulation and may exacerbate symptoms in COVID-19 patients [87, 88]. It is also suggested that the use of KKS and BK inhibitors can be considered a therapeutic approach for the patients with COVID-19, even prior to the administration of a COX inhibitor [79, 84].

## 5. Interaction between COVID-19, COX-2, and ACE2

Increased activity of COX-2 has been indicated in numerous experimental preparations such as lung injury induced by mechanical ventilation [89]. Inhibition of COX-2 is protective against lung damage caused by LPS in mice [90]. COX-2 inhibitors have antiviral and anti-inflammatory effects [91]. COX-2 inhibitor indomethacin has also been reported to be useful in treating the early stages of SARS-CoV-2 infection in dogs [92]. Moreover, the administration of nonsteroidal anti-inflammatory drugs (NSAIDs), ibuprofen and meloxicam, inhibits the production of proinflammatory cytokines and antibodies against SARS-CoV-2 infection in mice. However, it does not affect ACE2 expression, viral entry to cells, or viral replication in vitro or in vivo [93]. Another study reported that ibuprofen facilitates membrane ACE2 cleavage through the activation of ADAM-17 and prevents membrane-dependent virus entry into the cell by lowering the expression of TMPRSS2 [20]. Consequently, the antiviral effects of ibuprofen may be caused by its direct inhibitory effect on proinflammatory mediators, and indirectly, through its impact on the ACE2 cleavage within the cell membrane [20]. However, a decrease in cACE2 in patients with COVID-19 may augment the activity of the first arm of RAS and downstream COX-related inflammatory pathways, which must be explored in future studies on COVID-19 infection.

Data from two prospective cohort studies reported in Table 1 support the use of COX-2 inhibitors in patients with COVID-19. One study revealed that acute or chronic use of ibuprofen and other NSAIDs is not associated with worsening COVID-19 outcomes [94]. Celecoxib, a selective COX-2 inhibitor, is useful for the short-term treatment of patients with COVID-19 without worrying about major cardiovascular side effects [95]. Diclofenac is recommended as the best COX-2 inhibitor in the treatment of patients with COVID-19 at therapeutic doses [91]. Indomethacin is effective at reducing cough caused by BK during COVID-19 [96]. Meanwhile, a retrospective study found that NSAIDs, particularly selective COX-2 inhibitors, influence mild and severe COVID-19, while nonselective COX inhibitors have worse effects [97]. These findings indicate that the use of selective COX-2 inhibitors is essential for the treatment of patients with COVID-19. In addition, the use of NSAIDs in COVID-19 could reduce the natural host reactions necessary to fight viral infection and mask signs of infection [98].

## 6. Effects of Radiation Therapy on ACE, Bradykinin, and COX in COVID-19

Radiation therapy has been used to treat cancer and damaged tissue. However, the level, duration, and severity of damaged organs can predict the outcomes of the intervention. Basically, radiation acts as a double-edged sword. On the one hand, the anti-inflammatory effect of LDR has been identified in various experimental settings as well as in patients. However, even LDR can damage some organs like the heart, lungs, and kidneys. The monocyte adherence to the endothelium of the rat aorta increases by 1 to 24 hours after X-ray radiation with a dose of 2.5 Gy, whereas radiation with a dose of 7.5 Gy had no effect because of monocyte damage [99]. It has been demonstrated that radiation-induced heart disease is associated with the activity of the Ang II-aldosterone axis [100]. AT1 receptor antagonists and ACE inhibitors are effective in treating lung and kidney injuries after radiotherapy [21, 22]. Besides, LDR of 0.5 Gy with gamma-ray downregulates B2 receptors in HF-15 cells and consequently reduces inflammation [23]. These data suggest that the inflammatory or anti-inflammatory effects of B2 receptors are influenced by radiation levels and cell types. In addition, COX-2 can be activated by gamma radiation in PC-3 cells, dose dependently, which is inhibited by COX—an inhibitor of NS-398 [24].

A few studies have suggested the beneficial effects of low-dose radiation therapy in patients suffering from COVID-19 [101–103]. Two clinical trials with a small population revealed that 05–1.5 G of LDR led to low oxygen dependency of patients or no worsening of cytokine storm in COVID-19 patients, though extensive population studies are required for validation [104, 105] (Table 2). ARDS can be associated with a reduction in the number of leukocytes in blood, which can have a detrimental effect on the immune system. In addition, it takes 24 hours for radiation to have a maximum effect on macrophages. Therefore, it could not be recommended for treating COVID-19 patients with critical conditions [106].

## 7. Conclusion

Effective therapeutic approaches alongside global vaccination are needed to overcome such a challenging pandemic. The renin-angiotensin system appears to play a central role in the inflammatory response and cardiovascular disease in COVID-19 patients. Data from this review demonstrate that the timing of medication and disease severity are important for outcomes in patients with COVID-19. The use of BK and COX inhibitors can be recommended as a first step to prevent early inflammatory responses. Recombinant ACE2 can be administered to prevent increased viral internalization and replication, but several preclinical studies should be conducted before clinical trials in COVID-19 infection for final validation. Additionally, low-dose radiation may not be an option in severe COVID-19 patients. Moreover, combination therapy of recombinant ACE, BK inhibitors, and COX inhibitors should be evaluated in more animal models and large-scale clinical trials in the future. Of course, our study does not exclude multiple drug therapies for COVID-19 patients, but due to the wide spectrum of drug therapies, we investigated drugs that are somehow related to the renin-angiotensin system.

## Figures and Tables

**Table 1:** The effect of recombinant ACE2 in SARS-CoV-2 infection in vitro.

Study/subject	Drugs or exposure	Time of treatment	Outcome
Extracellular vesicles (EVs) exposing cACE2	1) SARS-CoV-2	First phase :1.5 h second phase: after 24 h	(i) Effective in vesicular viral trapping
	2) TMPRSS2		(ii) More efficient: cACE2 with TMPRSS2 [43]

Vero cells (monkey), human blood vessels, and kidney organoids	1) Clinical grade of hrsACE 2) and murine rsACE2: different concentrations	1 hour followed by washing, or 15 h without washing	Block the cell entry of SARS-CoV-2 [41]

Vero E6 cells (monkey) and kidney organoids	1) hrsACE2 APN01 (50–800 *μ*g/ml)	Kidney organoid: after 3 days Liver spheroids: after 15 h Measurement of cytotoxicity: after 24 h	Block the cell entry and replication of SARS-CoV-2 [40]
	2) Remdesivir (4–80 *μ*M)		

Renal cell line of HK2 (human) and Vero E6 cells (monkey)	1) Different concentrations of rACE2	3 days treatment	High concentration: inhibition of SARS-CoV-2 cell entry
			Physiologic concentration: increased viral cell entry [42]

**Table 2:** Drug administration in patients with COVID-19.

Study/Subject	Drugs and doses	Time (*T*) and duration (*D*) of treatments	Outcome
BK inhibition			

Man and woman (case-control)	1) 3 doses of 30 mg of icatibant (B receptor blocker of BK) by sc injection at 6-hour intervals	(i) T: at the onset of admission to hospital	A significant reduction in oxygen supplementation [46]
	2) Standard medications	(ii) D: 18 h (3 times each 6 hours)	

Man and woman (randomized trial protocol)	1) Icatibant 30 mg subcutaneously, 3 doses	(i) T: ≤12 days since the onset of the symptoms	Reducing the complications caused by COVID-19 pneumonia and duration of hospitalization [84]
	2) The inhibitor of C1e/kallikrein 20 U/kg, i.v on day 1 and 4	(ii) D: 4 days	
	3) Standard medications		

COX inhibition			
Man and woman (prospective cohort study)	1) Different NSAIDs	(i) T: acute: day1 chronic: before COVID-19 (ii) D: different	Mortality and hospital admission did not differ in acute and chronic treatments [85]
	2) Standard medications		

Man and woman (prospective cohort study)	1) Different NSAIDs	(i) T: different (ii) D: within 14 days before hospital admission	It was not associated with higher mortality or increased severity of disease [94]
	2) Standard medications		

Man and woman (retrospective cohort study)	1) Different NSAIDs	T: different	(i) Effective in mild disease (ii) COX-2 inhibitor was effective in severe disease (iii) Nonselective COX inhibitors had worse effects [107]
	2) Standard medications	D: different	

ACE2			
45-year-old woman (case report)	1) Soluble recombinant ACE2 (APN01), 0·4 mg/kg)	(i) T: 9 days after the onset of symptoms	ACE2 was well tolerated with no obvious side effects [97]
	2) Hydroxychloroquine, FIO2 of 70%, intubation, mechanical ventilation, cefuroxime, aztreonam	(ii) D: 5 minutes infusion twice a day lasting for 7 days	

Low-dose radiation			
Man and woman (clinical trial)	1) Whole lung irradiation 2) National protocol for the management of COVID-19	Radiation in a single fraction of 0.5 Gy	Encouraging results for oxygen dependency in 3 of 5 patients [104]

Man and woman (clinical trial)	1) Whole lung irradiation	A single-fraction radiation dose of 1.5 Gy	No worsening of the cytokine storm was observed in 4 of the 5 patients [105]

## Abbreviations

ADAM-17: A disintegrin and metalloprotease 17
Ang I: Angiotensin I
Ang II: Angiotensin II
Ang 1–7: Angiotensin 1–7
ACE: Angiotensin-converting enzyme
ACE2: Angiotensin-converting enzyme 2
AT1: Angiotensin receptor 1
AT2: Angiotensin receptor 2
ARDS: Acute respiratory distress syndrome
BP: Blood pressure
BK: Bradykinin
B1 receptors: BK receptor 1
B2 receptors: BK receptor 2
COVID-19: Coronavirus disease 2019
COX: Cyclooxygenase
DABK: Des-Arg-bradykinin
KKS: Kinin-kallikrein system
Gy: Gray
LDR: Low-dose radiation
NSAIDs: Nonsteroidal anti-inflammatory drugs
hrsACE2: Human recombinant soluble protein ACE2
RAS: Renin-angiotensin system
SARS-CoV: SARS coronavirus
TMPRSS2: Transmembrane protease, serine 2.

## References

[1] Bussard R. L., Busse L. W. (2018). Angiotensin II: a new therapeutic option for vasodilatory shock. *Therapeutics and Clinical Risk Management*.

[2] Santos R. A. S., Ferreira A. J., Verano-Braga T., Bader M. (2013). Angiotensin-converting enzyme 2, angiotensin-(1–7) and Mas: new players of the renin-angiotensin system. *Journal of Endocrinology*.

[3] Dhanachandra Singh K., Karnik S. S. (2017). Angiotensin receptors: structure, function, signaling and clinical applications. *Journal of cell signaling*.

[4] Kuba K., Imai Y., Penninger J. M. (2013). Multiple functions of angiotensin-converting enzyme 2 and its relevance in cardiovascular diseases. *Circulation Journal*.

[5] Huang C., Wang Y., Li X. (2020). Clinical features of patients infected with 2019 novel coronavirus in Wuhan, China. *The Lancet*.

[6] Jacoby D. S., Rader D. J. (2003). Renin-angiotensin system and atherothrombotic disease: from genes to treatment. *Archives of Internal Medicine*.

[7] Oudit G. Y., Kassiri Z., Patel M. P. (2007). Angiotensin II-mediated oxidative stress and inflammation mediate the age-dependent cardiomyopathy in ACE2 null mice. *Cardiovascular Research*.

[8] Ni W., Yang X., Yang D. (2020). Role of angiotensin-converting enzyme 2 (ACE2) in COVID-19. *Critical Care*.

[9] Zhang H., Penninger J. M., Li Y., Zhong N., Slutsky A. S. (2020). Angiotensin-converting enzyme 2 (ACE2) as a SARS-CoV-2 receptor: molecular mechanisms and potential therapeutic target. *Intensive Care Medicine*.

[10] Ferrara F., Vitiello A. (2021). Scientific hypothesis for treatment of COVID-19’s lung lesions by adjusting ACE/ACE2 imbalance. *Cardiovascular Toxicology*.

[11] Wiese O. J., Allwood B. W., Zemlin A. E. (2020). COVID-19 and the renin-angiotensin system (RAS): a spark that sets the forest alight?. *Medical Hypotheses*.

[12] Wan Y., Shang J., Graham R., Baric R. S., Li F. (2020). Receptor recognition by the novel coronavirus from wuhan: an analysis based on decade-long structural studies of SARS coronavirus. *Journal of Virology*.

[13] Walls A. C., Park Y. J., Tortorici M. A., Wall A., McGuire A. T., Veesler D. (2020). Structure, function, and antigenicity of the SARS-CoV-2 spike glycoprotein. *Cell*.

[14] Harmer D., Gilbert M., Borman R., Clark K. L. (2002). Quantitative mRNA expression profiling of ACE 2, a novel homologue of angiotensin converting enzyme. *FEBS Letters*.

[15] Jia H. P., Look D. C., Tan P. (2009). Ectodomain shedding of angiotensin converting enzyme 2 in human airway epithelia. *American Journal of Physiology - Lung Cellular and Molecular Physiology*.

[16] Li M.-Y., Li L., Zhang Y., Wang X.-S. (2020). Expression of the SARS-CoV-2 cell receptor gene ACE2 in a wide variety of human tissues. *Infectious diseases of poverty*.

[17] Han T., Kang J., Li G., Ge J., Gu J. (2020). Analysis of 2019-nCoV receptor ACE2 expression in different tissues and its significance study. *Annals of Translational Medicine*.

[18] Zhang H., Li H.-B., Lyu J.-R. (2020). Specific ACE2 expression in small intestinal enterocytes may cause gastrointestinal symptoms and injury after 2019-nCoV infection. *International Journal of Infectious Diseases*.

[19] Donoghue M., Hsieh F., Baronas E. (2000). A novel angiotensin-converting enzyme-related carboxypeptidase (ACE2) converts angiotensin I to angiotensin 1-9. *Circulation Research*.

[20] Smart L., Fawkes N., Goggin P. (2020). A narrative review of the potential pharmacological influence and safety of ibuprofen on coronavirus disease 19 (COVID-19), ACE2, and the immune system: a dichotomy of expectation and reality. *Inflammopharmacology*.

[21] Moulder E., Cohen J., Fish B. L., Cohen E. P. (1998). Angiotensin II receptor antagonists in the treatment and prevention of radiation nephropathy. *International Journal of Radiation Biology*.

[22] Moulder J. E., Fish B. L., Cohen E. P. (2007). Treatment of radiation nephropathy with ACE inhibitors and AII type-1 and type-2 receptor antagonists. *Current Pharmaceutical Design*.

[23] Micke P., Blaukat A., Micke O. (2003). Effect of cobalt-60 irradiation on bradykinin B2 receptor expression on human HF-15 cells.

[24] Steinauer K. K., Gibbs I., Ning S., French J. N., Armstrong J., Knox S. J. (2000). Radiation induces upregulation of cyclooxygenase-2 (COX-2) protein in PC-3 cells. *International Journal of Radiation Oncology, Biology, Physics*.

[25] Hamming I., Timens W., Bulthuis M., Lely A., Navis G., van Goor H. (2004). Tissue distribution of ACE2 protein, the functional receptor for SARS coronavirus. A first step in understanding SARS pathogenesis. *The Journal of Pathology*.

[26] Kuhn J. H., Li W., Choe H., Farzan M. (2004). Angiotensin-converting enzyme 2: a functional receptor for SARS coronavirus. *Cellular and Molecular Life Sciences: CM*.

[27] Li W., Moore M. J., Vasilieva N. (2003). Angiotensin-converting enzyme 2 is a functional receptor for the SARS coronavirus. *Nature*.

[28] Wong S. K., Li W., Moore M. J., Choe H., Farzan M. (2004). A 193-amino acid fragment of the SARS coronavirus S protein efficiently binds angiotensin-converting enzyme 2. *Journal of Biological Chemistry*.

[29] Li W., Zhang C., Sui J. (2005). Receptor and viral determinants of SARS-coronavirus adaptation to human ACE2. *The EMBO Journal*.

[30] Gu J., Gong E., Zhang B. (2005). Multiple organ infection and the pathogenesis of SARS. *Journal of Experimental Medicine*.

[31] Kuba K., Imai Y., Rao S. (2005). A crucial role of angiotensin converting enzyme 2 (ACE2) in SARS coronavirus–induced lung injury. *Nature Medicine*.

[32] Dales N. A., Gould A. E., Brown J. A. (2002). Substrate-based design of the first class of angiotensin-converting enzyme-related carboxypeptidase (ACE2) inhibitors. *Journal of the American Chemical Society*.

[33] Huang L., Sexton D. J., Skogerson K. (2003). Novel peptide inhibitors of angiotensin-converting enzyme 2. *Journal of Biological Chemistry*.

[34] Osman I. O., Melenotte C., Brouqui P. (2021). Expression of ACE2, soluble ACE2, angiotensin I, angiotensin II and angiotensin-(1–7) is modulated in COVID-19 patients. *Frontiers in Immunology*.

[35] Chen J., Jiang Q., Xia X. (2020). Individual variation of the SARS-CoV-2 receptor ACE2 gene expression and regulation. *Aging Cell*.

[36] Hoffmann M., Kleine-Weber H., Schroeder S. (2020). SARS-CoV-2 cell entry depends on ACE2 and TMPRSS2 and is blocked by a clinically proven protease inhibitor. *Cell*.

[37] Heurich A., Hofmann-Winkler H., Gierer S., Liepold T., Jahn O., Pöhlmann S. (2014). TMPRSS2 and ADAM17 cleave ACE2 differentially and only proteolysis by TMPRSS2 augments entry driven by the severe acute respiratory syndrome coronavirus spike protein. *Journal of Virology*.

[38] Zipeto D., Palmeira JdF., Argañaraz G. A., Argañaraz E. R. (2020). ACE2/ADAM17/TMPRSS2 interplay may Be the main risk factor for COVID-19. *Frontiers in Immunology*.

[39] Lambert D. W., Yarski M., Warner F. J. (2005). Tumor necrosis factor-alpha convertase (ADAM17) mediates regulated ectodomain shedding of the severe-acute respiratory syndrome-coronavirus (SARS-CoV) receptor, angiotensin-converting enzyme-2 (ACE2). *Journal of Biological Chemistry*.

[40] Monteil V., Dyczynski M., Lauschke V. M. (2021). Human soluble ACE2 improves the effect of remdesivir in SARS-CoV-2 infection. *EMBO Molecular Medicine*.

[41] Monteil V., Kwon H., Prado P. (2020). Inhibition of SARS-CoV-2 infections in engineered human tissues using clinical-grade soluble human ACE2. *Cell*.

[42] Yeung M. L., Teng J. L. L., Jia L. (2021). Soluble ACE2-mediated cell entry of SARS-CoV-2 via interaction with proteins related to the renin-angiotensin system. *Cell*.

[43] Cocozza F., Névo N., Piovesana E. (2020). Extracellular vesicles containing ACE2 efficiently prevent infection by SARS-CoV-2 Spike protein-containing virus. *Journal of Extracellular Vesicles*.

[44] Khan A., Benthin C., Zeno B. (2017). A pilot clinical trial of recombinant human angiotensin-converting enzyme 2 in acute respiratory distress syndrome. *Critical Care*.

[45] Zoufaly A., Poglitsch M., Aberle J. H. (2020). Human recombinant soluble ACE2 in severe COVID-19. *The Lancet Respiratory Medicine*.

[46] Batlle D., Wysocki J., Satchell K. (2020). Soluble angiotensin-converting enzyme 2: a potential approach for coronavirus infection therapy?. *Clinical Science*.

[47] Krishnamurthy S., Lockey R. F., Kolliputi N. (2021). Soluble ACE2 as a potential therapy for COVID-19. *American Journal of Physiology—Cell Physiology*.

[48] Jia H., Neptune E., Cui H. (2021). Targeting ACE2 for COVID-19 therapy: opportunities and challenges. *American Journal of Respiratory Cell and Molecular Biology*.

[49] Kumar P., Sah A. K., Tripathi G. (2021). Role of ACE2 receptor and the landscape of treatment options from convalescent plasma therapy to the drug repurposing in COVID-19. *Molecular and Cellular Biochemistry*.

[50] Kragstrup T. W., Singh H. S., Grundberg I. (2021). Plasma ACE2 predicts outcome of COVID-19 in hospitalized patients. *PLoS One*.

[51] Maza M. D. C., Úbeda M., Delgado P. (2022). ACE2 serum levels as predictor of infectability and outcome in COVID-19. *Frontiers in Immunology*.

[52] Liu Y., Yang Y., Zhang C. (2020). Clinical and biochemical indexes from 2019-nCoV infected patients linked to viral loads and lung injury. *Science China Life Sciences*.

[53] Zhang P., Zhu L., Cai J. (2020). Association of inpatient use of angiotensin-converting enzyme inhibitors and angiotensin II receptor blockers with mortality among patients with hypertension hospitalized with COVID-19. *Circulation Research*.

[54] Meng J., Xiao G., Zhang J. (2020). Renin-angiotensin system inhibitors improve the clinical outcomes of COVID-19 patients with hypertension. *Emerging Microbes & Infections*.

[55] Liu Y., Huang F., Xu J. (2020). Anti-hypertensive Angiotensin II receptor blockers associated to mitigation of disease severity in elderly COVID-19 patients. *medRxiv*.

[56] Mehta N., Kalra A., Nowacki A. S. (2020). Association of use of angiotensin-converting enzyme inhibitors and angiotensin II receptor blockers with testing positive for coronavirus disease 2019 (COVID-19). *JAMA Cardiology*.

[57] Wang Z., Zhang D., Wang S. (2020). A retrospective study from 2 centers in China on the effects of continued use of angiotensin-converting enzyme inhibitors and angiotensin II receptor blockers in patients with hypertension and COVID-19. *Medical Science Monitor: International Medical Journal of Experimental and Clinical Research*.

[58] Mancia G., Rea F., Ludergnani M., Apolone G., Corrao G. (2020). Renin–angiotensin–aldosterone system blockers and the risk of Covid-19. *New England Journal of Medicine*.

[59] Bravi F., Flacco M. E., Carradori T. (2020). Predictors of severe or lethal COVID-19, including angiotensin converting enzyme inhibitors and angiotensin II receptor blockers, in a sample of infected Italian citizens. *PLoS One*.

[60] de Abajo F. J., Rodríguez-Martín S., Lerma V. (2020). Use of renin–angiotensin–aldosterone system inhibitors and risk of COVID-19 requiring admission to hospital: a case-population study. *The Lancet*.

[61] Jung S.-Y., Choi J. C., You S.-H., Kim W.-Y. (2020). Association of renin-angiotensin-aldosterone system inhibitors with coronavirus disease 2019 (COVID-19)-related outcomes in Korea: a nationwide population-based cohort study. *Clinical Infectious Diseases*.

[62] Spaccarotella C., Mazzitelli M., Migliarino S. (2021). Therapy with RAS inhibitors during the COVID-19 pandemic. *Journal of Cardiovascular Medicine*.

[63] Rao R., Husain A., Bharti A. C., Kashyap M. K. (2019). Discovery of a novel connecting link between renin-angiotensin system and cancer in barrett’s esophagus by proteomic screening. *Proteomics - Clinical Applications*.

[64] Ahmad M., Zeitlin I. J., Parratt J. R., Pitt A. R. (2006). Degradation of bradykinin, a cardioprotective substance, during a single passage through isolated rat-heart. *Archives of Pharmacal Research*.

[65] Tikellis C., Thomas M. C. (2012). Angiotensin-converting enzyme 2 (ACE2) is a key modulator of the renin angiotensin system in health and disease. *International Journal of Peptides*.

[66] Vickers C., Hales P., Kaushik V. (2002). Hydrolysis of biological peptides by human angiotensin-converting enzyme-related carboxypeptidase. *Journal of Biological Chemistry*.

[67] Sodhi C. P., Wohlford-Lenane C., Yamaguchi Y. (2018). Attenuation of pulmonary ACE2 activity impairs inactivation of des-Arg9 bradykinin/BKB1R axis and facilitates LPS-induced neutrophil infiltration. *American Journal of Physiology—Lung Cellular and Molecular Physiology*.

[68] Yu H., Carretero O. A., Juncos L. A., Garvin J. L. (1998). Biphasic effect of bradykinin on rabbit afferent arterioles. *Hypertension*.

[69] McLean P. G., Perretti M., Ahluwalia A. (2000). Kinin B(1) receptors and the cardiovascular system: regulation of expression and function. *Cardiovascular Research*.

[70] Bélichard P., Loillier B., Paquet J. L., Luccarini J. M., Pruneau D. (1996). Haemodynamic and cardiac effects of kinin B1 and B2 receptor stimulation in conscious instrumented dogs. *British Journal of Pharmacology*.

[71] Greco A. J., Master R. G., Fokin A., Baber S. R., Kadowitz P. J. (2006). Angiotensin-(1–7) potentiates responses to bradykinin but does not change responses to angiotensin I. *Canadian Journal of Physiology and Pharmacology*.

[72] Raffai G., Khang G., Vanhoutte P. M. (2014). Angiotensin-(1–7) augments endothelium-dependent relaxations of porcine coronary arteries to bradykinin by inhibiting angiotensin-converting enzyme 1. *Journal of Cardiovascular Pharmacology*.

[73] Anton E. L., Fernandes D., Assreuy J., Silva-Santos J. E. (2019). Bradykinin increases BP in endotoxemic rat: functional and biochemical evidence of angiotensin II AT(1)/bradykinin B(2) receptor heterodimerization. *British Journal of Pharmacology*.

[74] Sharma J. (2010). *Activation of the Bradykinin System by Angiotensin-Converting Enzyme Inhibitors*.

[75] Tang Z., Wang Z., Hu Z., Zhang M., Li L., Li B. (2016). The role of bradykinin in lung ischemia-reperfusion injury in a rat lung transplantation model. *Acta Cirurgica Brasileira*.

[76] Broadley K. J., Blair A. E., Kidd E. J., Bugert J. J., Ford W. R. (2010). Bradykinin-induced lung inflammation and bronchoconstriction: role in parainfluenze-3 virus-induced inflammation and airway hyperreactivity. *Journal of Pharmacology and Experimental Therapeutics*.

[77] Lariccia V., Magi S., Serfilippi T., Toujani M., Gratteri S., Amoroso S. (2020). Challenges and opportunities from targeting inflammatory responses to SARS-CoV-2 infection: a narrative review. *Journal of Clinical Medicine*.

[78] Garvin M. R., Alvarez C., Miller J. I. (2020). A mechanistic model and therapeutic interventions for COVID-19 involving a RAS-mediated bradykinin storm. *Elife*.

[79] Roche J. A., Roche R. (2020). A hypothesized role for dysregulated bradykinin signaling in COVID-19 respiratory complications. *The FASEB Journal*.

[80] Bryant J., Shariat-Madar Z. (2009). Human plasma kallikrein-kinin system: physiological and biochemical parameters. *Cardiovascular and Hematological Agents in Medicinal Chemistry*.

[81] Chichorro J. G., Lorenzetti B. B., Zampronio A. R. (2004). Involvement of bradykinin, cytokines, sympathetic amines and prostaglandins in formalin-induced orofacial nociception in rats. *British Journal of Pharmacology*.

[82] Bujalska M., Makulska-Nowak H. (2009). Bradykinin receptor antagonists and cyclooxygenase inhibitors in vincristine- and streptozotocin-induced hyperalgesia. *Pharmacological Reports*.

[83] Sidarta-Oliveira D., Jara C. P., Ferruzzi A. J. (2020). SARS-CoV-2 receptor is co-expressed with elements of the kinin–kallikrein, renin–angiotensin and coagulation systems in alveolar cells. *Scientific Reports*.

[84] van de Veerdonk F. L., Kouijzer I. J. E., de Nooijer A. H. (2020). Outcomes associated with use of a kinin B2 receptor antagonist among patients with COVID-19. *JAMA Network Open*.

[85] Mansour E., Bueno F. F., de Lima-Júnior J. C. (2021). Evaluation of the efficacy and safety of icatibant and C1 esterase/kallikrein inhibitor in severe COVID-19: study protocol for a three-armed randomized controlled trial. *Trials*.

[86] Mohammed El Tabaa M., Mohammed El Tabaa M. (2020). Targeting Neprilysin (NEP) pathways: a potential new hope to defeat COVID-19 ghost. *Biochemical Pharmacology*.

[87] Goldin C. J., Vázquez R., Polack F. P., Alvarez-Paggi D. (2020). Identifying pathophysiological bases of disease in COVID-19. *Translational Medicine Communications*.

[88] Dicpinigaitis P. V. (2006). Angiotensin-converting enzyme inhibitor-induced cough: ACCP evidence-based clinical practice guidelines. *Chest*.

[89] Robertson J. A., Sauer D., Gold J. A., Nonas S. A. (2012). The role of cyclooxygenase-2 in mechanical ventilation-induced lung injury. *American Journal of Respiratory Cell and Molecular Biology*.

[90] Liou C. J., Lai Y. R., Chen Y. L., Chang Y. H., Li Z. Y., Huang W. C. (2016). Matrine attenuates COX-2 and ICAM-1 expressions in human lung epithelial cells and prevents acute lung injury in LPS-induced mice. *Mediators of Inflammation*.

[91] Verrall G. M. (2020). Scientific rationale for a bottom-up approach to target the host response in order to try and reduce the numbers presenting with adult respiratory distress syndrome associated with COVID-19. Is there a role for statins and COX-2 inhibitors in the prevention and early treatment of the disease?. *Frontiers in Immunology*.

[92] Amici C., Caro A. D., Ciucci A. (2006). Indomethacin has a potent antiviral activity against SARS coronavirus. *Antiviral Therapy*.

[93] Chen J. S., Alfajaro M. M., Chow R. D. (2021). Nonsteroidal anti-inflammatory drugs dampen the cytokine and antibody response to SARS-CoV-2 infection. *Journal of Virology*.

[94] Abu Esba L. C., Alqahtani R. A., Thomas A., Shamas N., Alswaidan L., Mardawi G. (2021). Ibuprofen and NSAID use in COVID-19 infected patients is not associated with worse outcomes: a prospective cohort study. *Infectious diseases and therapy*.

[95] Baghaki S., Yalcin C. E., Baghaki H. S., Aydin S. Y., Daghan B., Yavuz E. (2020). COX2 inhibition in the treatment of COVID-19: review of literature to propose celecoxib repositioning for randomized controlled studies. *International Journal of Infectious Diseases*.

[96] Alkotaji M., Al-Zidan R. N. (2021). Indomethacin: can it counteract bradykinin effects in COVID-19 patients?. *Current Pharmacology Reports*.

[97] Reese J. T., Coleman B., Chan L. (2021). Cyclooxygenase inhibitor use is associated with increased COVID-19 severity. *medRxiv*.

[98] Robb C. T., Goepp M., Rossi A. G., Yao C. (2020). Non-steroidal anti-inflammatory drugs, prostaglandins, and COVID-19. *British Journal of Pharmacology*.

[99] Korystova A. F., Kublik L. N., Levitman M. K., Samokhvalova T. V., Shaposhnikova V. V., Korystov Y. N. (2018). Ionizing radiation enhances activity of angiotensin-converting enzyme in rat aorta. *Bulletin of Experimental Biology and Medicine*.

[100] Wu R., Zeng Y. (2009). Does angiotensin II-aldosterone have a role in radiation-induced heart disease?. *Medical Hypotheses*.

[101] Mehdizadeh A., Bevelacqua J., Mortazavi S., Mortazavi S. (2020). COVID-19: introducing low dose radiation as an effective treatment for pneumonia that shouldn’t induce selective pressure and new mutations. *Journal of Biomedical Physics & Engineering*.

[102] Salomaa S., Cardis E., Bouffler S. D., Atkinson M. J., Hamada N. (2020). Low dose radiation therapy for COVID-19 pneumonia: is there any supportive evidence?. *International Journal of Radiation Biology*.

[103] Sanmamed N., Alcantara P., Cerezo E. (2021). Low-dose radiation therapy in the management of coronavirus disease 2019 (COVID-19) pneumonia (LOWRAD-Cov19): preliminary report. *International Journal of Radiation Oncology, Biology, Physics*.

[104] Ameri A., Rahnama N., Bozorgmehr R. (2020). Low-dose whole-lung irradiation for COVID-19 pneumonia: short course results. *International Journal of Radiation Oncology, Biology, Physics*.

[105] Hess C. B., Buchwald Z. S., Stokes W. (2020). Low-dose whole-lung radiation for COVID-19 pneumonia: planned day 7 interim analysis of a registered clinical trial. *Cancer*.

[106] Venkatesulu B. P., Lester S., Hsieh C.-E. (2021). Low-dose radiation therapy for COVID-19: promises and pitfalls. *JNCI Cancer Spectrum*.

[107] Drake T. M., Fairfield C. J., Pius R. (2021). Non-steroidal anti-inflammatory drug use and outcomes of COVID-19 in the ISARIC Clinical Characterisation Protocol UK cohort: a matched, prospective cohort study. *The Lancet Rheumatology*.

